# Bioactive Compounds and Valorization of Coffee By-Products from the Origin: A Circular Economy Model from Local Practices in Zongolica, Mexico

**DOI:** 10.3390/plants13192741

**Published:** 2024-09-30

**Authors:** Emanuel Bojórquez-Quintal, Damián Xotlanihua-Flores, Loretta Bacchetta, Gianfranco Diretto, Oliviero Maccioni, Sarah Frusciante, Luis M. Rojas-Abarca, Esteban Sánchez-Rodríguez

**Affiliations:** 1 CONAHCYT, Laboratorio de Análisis y Diagnóstico del Patrimonio, El Colegio de Michoacán, Cerro de Nahuatzen 85, La Piedad 59379, Michoacán, Mexico; 2 Ingeniería en Desarrollo Comunitario, Instituto Tecnológico Superior de Zongolica, Km 4 Carretera a la Compañía S/N, Tepetlitlanapa, Zongolica 95005, Veracruz, Mexico; damian_xotlanihua_pd314@zongolica.tecnm.mx; 3 Regenerative Circular Bioeconomy Laboratory, AGROS Division, SSPT Department, ENEA Casaccia, Via Anguillarese 301, 00123 Rome, Italy; loretta.bacchetta@enea.it (L.B.); oliviero.maccioni@enea.it (O.M.); 4 GREEN Biotechnology Laboratory, BIOAG Division, SSPT Department, ENEA Casaccia, Via Anguillarese 301, 00123 Rome, Italy; gianfranco.diretto@enea.it (G.D.); sarah.frusciante@enea.it (S.F.); 5 Laboratorio de Análisis y Diagnóstico del Patrimonio, El Colegio de Michoacán, Cerro de Nahuatzen 85, La Piedad 59379, Michoacán, Mexico; labarca@colmich.edu.mx (L.M.R.-A.); esanchez@colmich.edu.mx (E.S.-R.)

**Keywords:** bioactive compounds, foodoma, foodomics, coffee agroecology, coffee by-products, waste valorization, rural practices, HPLC-ESI-HRMS, DART-MS, SEM-EDS microanalysis

## Abstract

The by-products of green coffee processing are rich in compounds that can be recycled for their possible use in the production of beverages, fertilizers and weed control in production areas. The objective of this work was to identify the organic and inorganic bioactive compounds of green coffee and the coffee by-products related to the production of origin, such as dried cascara (skin-pulp), parchment and silverskin (unroasted), in order to investigate the role their biomolecules may have in reuse through practices and local knowledge, not yet valued. The metabolomic profile by HPLC-ESI-HRMS of the aqueous extract of the dried cascara highlighted 93 non-volatile molecules, the highest number reported for dried cascara. They belong to groups of organic acids (12), alkaloids (5), sugars (5), fatty acids (2), diglycerides (1), amino acids (18), phospholipids (7), vitamins (5), phenolic acids (11), flavonoids (8), chlorogenic acids (17), flavones (1) and terpenes (1). For the first time, we report the use of direct analysis in real-time mass spectrometry (DART-MS) for the identification of metabolites in aqueous extracts of dried cascara, parchment, silverskin and green coffee. The DART analysis mainly showed the presence of caffeine and chlorogenic acids in all the extracts; additionally, sugar adducts and antioxidant compounds such as polyphenols were detected. The mineral content (K, Ca, P, S, Mg and Cl) by EDS spectrometry in the by-products and green coffee showed a relatively high content of K in the dried cascara and green coffee, while Ca was detected in double quantity in the silverskin. These metabolomic and mineral profile data allow enhancement of the link between the quality of green coffee and its by-products and the traditional local practices in the crop-growing area. This consolidates the community’s experience in reusing by-products, thereby minimizing the impact on the environment and generating additional income for coffee growers’ work, in accordance with the principles of circular economy and bioeconomy.

## 1. Introduction

Coffee beans cannot be grown everywhere in the world. There is a natural line called the green coffee belt, between the Tropics of Cancer and Capricorn, where almost eighty countries can potentially be coffee suppliers [1]. Production is concentrated in developing countries, where coffee represents a considerable share of export earnings and provides a key source of livelihood for more than 25 million families; however, green coffee is exported at an early stage of the value chain, providing little added value in producing countries [2]. In Latin America, the main exporting countries are Brazil, Colombia, Guatemala, Honduras, Peru, Mexico and Costa Rica [1].

Coffee is the second most traded product, only surpassed by oil. World green coffee production in 2023/2024 has been estimated at 178.0 million bags/60 Kg, 57.4% of which is Arabica coffee and 42.6% is Robusta coffee [3]. However, the supply chain generates exploitation and social and economic inequality in producing countries (mainly developing) and a wide variety of derivatives from coffee processing (pulp, mucilage, parchment, dried husk and 80% of the cherry) in production countries and from the roasting industry (silverskin and spent coffee grounds, approximately 14.4%) in consumption countries [4,5]. These by-products have a negative economic, social, ecological and environmental impact. In 2023/2024, we estimate close to 840.16 million bags/60 kg of by-products and waste in producing and consuming countries due to global coffee production [3].

Considering the volatility of international coffee prices, environmental factors such as climate change, continuous pests and economic factors that directly affect production costs (which may be higher than the final sale price of coffee), organic and specialty coffee markets, through the purchase of quality green coffee [1], as well as the valorization and trade of by-products, could be beneficial for coffee-producing families and the global coffee industry [4].

A proposal to expand the usefulness and added value of green coffee and by-products from primary coffee processing is to increase the knowledge of their organic and inorganic chemical composition [6,7]. Green coffee, by-products and residues are sources of macromolecules (carbohydrates, proteins, pectin) and bioactive compounds [6,7,8], which can be extracted and reused to develop new food, nutraceutical, agricultural, cosmetic and pharmaceutical products at low cost and according to the principles of bioeconomy and circular economy promoted by the FAO [2,6]. Bioactive compounds include primary and secondary metabolites, the latter mainly being flavonoids, terpenoids, polyphenols, alkaloids such as caffeine, saponins, bioactive peptides and unsaturated fatty acids. Vitamins, pigments, carotenoids, amino acids, sugars and minerals, due to their pharmacological effects, can be included as bioactive compounds [9,10].

In the last decade, several studies and publications have focused on reusing, recycling and repurposing coffee by-products and waste [6]. While there is a marked tendency to develop bioproducts and promising sustainable development opportunities in coffee-consuming countries (e.g., European Union countries) [8,11,12,13], potential realistic applications in producing countries have not yet been considered [4,14]. Although the importance of strategies for revaluing by-products from primary coffee processing has been highlighted [6], there are few studies on local practices and traditional knowledge of coffee-producing families; only traditional beverages and tea-type infusions have been reported [15,16].

On the other land, the chemical composition of coffee by-products and waste has been studied by different authors [6,8,11,13,17]. However, a more in-depth knowledge of the compositional characteristics of waste matrices from the coffee production chain is necessary to encourage their efficient reuse. For instance, despite its potential uses and its designation as a novel food in the European Union [4,18], dried husk (pulp or cascara) is among the least-characterized coffee cherry by-products, in terms of its polar and semi-polar bioactive molecules; recent studies focused mainly on its volatile molecules and aromatic characteristics [15,16]. Parchment and silverskin (unroasted) are primary processing by-products that have received little attention; therefore, they are poorly studied and valued for their chemical composition. The chemical profile of green coffee beans in relation to agroecological and local practices has not been well explored. This profile could be a fingerprint of quality, circularity and marketing at a better price in specialty markets [1].

**Figure 1:**
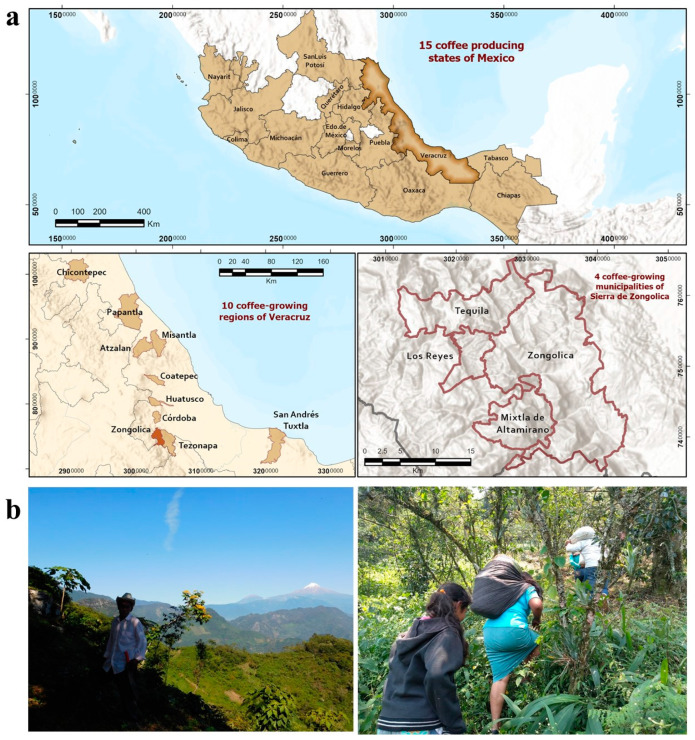
Macrolocation and coffee landscape of the Sierra de Zongolica, Veracruz, Mexico. (**a**) Location of the coffee-growing regions of the State of Veracruz and coffee-growing municipalities of the Sierra de Zongolica. (**b**) Coffee landscape observed from a panoramic view from southwest to northwest. Interaction between the producer, coffee grower and the coffee plantation, and the slopes with shade and light. Maps and photos by DXF and JMR. Geographic maps based on INEGI data and prepared with the ArcGis Pro v3.3 program.

Mexico is a leader in certified organic coffee production, stands out in specialty coffee production, and has the potential to generate foreign currency and jobs, contributing to the recovery and strengthening of sustainability in rural societies, natural agroecosystems and agri-food systems [1]. In Mexico, coffee cultivation is the livelihood of small producers belonging to 32 ethnic groups in 15 producing states (Figure 1). However, agroecological practices in coffee cultivation and the reuse of by-products still have no added value for small producers. The final purchase price of this coffee (in parchment coffee or green coffee) lacks traceability and marketing. In addition, the diversification of income from the sale of the by-products of primary production lacks national acceptance and marketing. In this sense, metabolomics and mineral profiling can improve the link between the quality of green coffee and primary coffee by-products and the traditional cultivation area and its local practices. In addition, they can favor the valorization and reuse of coffee by-products for the development of new functional foods and for non-food applications in coffee-producing regions.

This work aimed to increase the knowledge on the pool of bioactive compounds, organic and inorganic, present in coffee products (green coffee) and by-products (dried cascara, parchment and unroasted silverskin) generated in the traditional area of coffee cultivation. In addition, the use and awareness of local waste reuse activities and practices were considered in a view of local circular economy and bioeconomy. Composting and fertilization activities, production of infusions and weed control in the Sierra de Zongolica are described and the bioactive compounds identified. We took this area as a main example and as a case study for different reasons: it has a thousand-year history of use and cultivation of coffee, it is considered a region that produces high quality of coffee, it has wide local traditions linked with coffee, and finally, it is an example of sustainable cultivation management [1]. Thus, our advances in chemical characterization could efficiently address the biological applications of coffee by-products with useful implications also in other coffee cultivation contexts.

## 2. Results and Discussion

### 2.1. Traditional and Local Practices of Reuse of By-Products in Sierra de Zongolica

**Figure 2:**
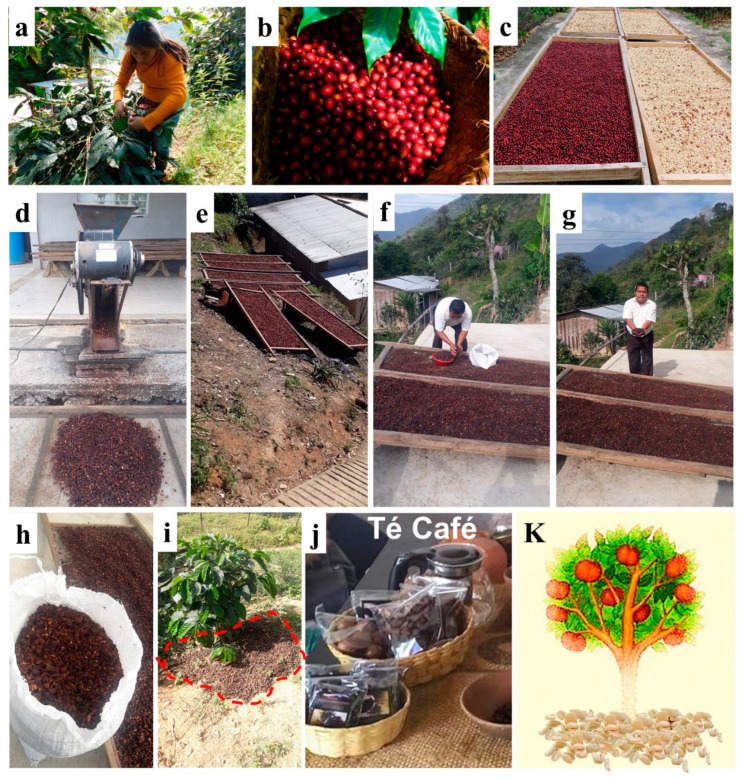
Processing and reuse of coffee by-products by local practices in the Sierra de Zongolica, Veracruz, Mexico. (**a**,**b**) Harvesting and sorting of ripe coffee cherries. (**c**) Products from dry and wet processing of coffee cherries, dried coffee cherries and parchment coffee. (**d**–**h**) Processing, drying and collection of dry coffee husks after dry and wet processing. (**i**–**k**) Reuse of by-products (dried cascara/husk and parchment) by local practices as free fertilizer, tea-type beverages and weed control. Photos by DXF.

The high mountain area of the Sierra de Zongolica, in Veracruz, stands out as a region where the organic cultivation of high-altitude coffee is practiced over 1000 m above sea level, under shade, in agro-environmental conditions of difficult access (Figure 1). In this area, the agroecological practices such as the traditional, rustic or natural polyculture, milpa system and local habits allow indigenous peoples to face the environmental and economic crises of Mexican coffee growing. The production of Arabica coffees in Zongolica is of high quality, without the use of pesticides, fertilizers and other chemicals. Coffee is grown using agricultural practices adapted to local ecological contexts, associated with food crops for self-consumption, which protect biodiversity and biocultural diversity and maintain low dependence on external inputs. The cherry harvest is made by manual selection of ripe fruits with the participation of women and family members (Figure 2).

However, traditional agricultural systems face major challenges associated with environmental deterioration, climate instability, sociocultural changes, asymmetric trade relations and unfavorable policies. In fact, agroecological practices in coffee cultivation continue to have no added value for small producers; the final purchase price of this coffee (in parchment coffee or green coffee) lacks traceability and marketing, and the added value (80%) remains for the benefit of companies [1]. Due to this situation, a group of 20 coffee-growing families from the Nahuatl communities of Tlecuaxco and Poxcautla began a process of diversifying their economic income through coffee production, which is supported by the reuse of by-products generated by the wet and dry processing of cherries, such as dry cascara, honey water (mucilage washing water) and parchment (Figure 2).

In the Sierra de Zongolica, to produce one kilogram (kg) of green coffee from the wet process, five kg of fresh cherries are needed, and four kg of dried cascara (dried husk) are obtained as a by-product. Of this dried cascara generated during the primary processing of coffee, 40% is reused as free fertilizer added to the soil (Figure 2), and 55% is not useful. The remaining 5% of the dried cascara is traditionally consumed in the Sierra de Zongolica as an infusion similar to tea or sold to prepare tisane. Also, the dried coffee flowers are collected for tea-type drinks. The dried cascara from wet and dry processing is also reused as compost, and the honey water from washings is used as liquid fertilizer. Other traditional uses of the cascara are as animal feed [6,14] or as a substrate for the growth of edible mushrooms. The cascara (skin and pulp) has potential use as a nutritional and functional food additive in baking, the production of cookies, fruity-flavored purees and jams and cold drinks [4]. Recently, based on traditional food declarations and requests from third countries, the European Food Safety Authority (EFSA) has assessed and considered the possibility of marketing dried cascara as a novel food in the European Union market [4,18]. This fact increases the possibility of diversifying economic income from the sale of dried coffee cascara, similar to the trade of specialty coffees to the European market [1]. In Switzerland, dried coffee cherry pulp has been used to produce a refreshing, functional and stimulating beverage called Cascara, also known as coffee cherry tea [11]. In addition, a successful ready-to-drink tisane-like product made in Hawaii from wet processed coffee skin and pulp is marketed as “Kona Red”. Others traditional beverages from coffee cherries have been consumed in Yemen as “Qishr” and Ethiopia as “Hashara” [4]. In Latin America, the traditional consumption of dried cascara is in “tea-type drinks” [4,16].

Parchment, another by-product generated by the commercialization of green coffee, can be reused as a weed control around fruit trees (Figure 2k). In addition, due to its capacity as an absorbent material, it favors the retention of fertilizers, manures and water. Parchment is a lignocellulosic material with hygroscopic and fat-absorbing properties. It is used in poultry farming as poultry litter and as a composite construction material. Parchment and silverskin (unroasted) are little studied or valued. Both by-products are bulky, but their low humidity facilitates their storage and direct use [19]. Extracts of parchment and silverskin can be used as antioxidants and antifungal additives due to their polyphenolic compounds [20]. The traditional use of parchment as a weed controller and poultry litter is likely due to the herbicidal and antifungal activity of the bioactive compounds present in this by-product. Other reported uses of parchment are as a food preservative and biodegradable packaging material [19]. Unroasted silverskin has no use, unlike roasted silverskin, which is a food ingredient, thickener and colorant [14] and has applications in infusions [17]. Silverskin is also an input for edible mushroom cultivation, and its composting has been suggested to improve the physical and chemical properties of the soil.

Green coffee beans are directly related to the geographical area, their cultivation and processing. In addition, their genetic load provides information on the origin of production, and their chemical footprint influences the quality of the roasted coffee in the cup [4]. Green coffee and defective beans could be used for food supplements [13] or green coffee drinks, due to their antioxidant activity and bioactive components [21]. The chemical profile of the green coffee bean by agroecological practices and local practices has not been explored or valued; it could be a fingerprint of quality, circularity and commercialization. For example, the German market, unlike the American market, represents a good opportunity, especially for quality coffees from the Arabica species (*Coffea arabica*) such as Typica, Bourbon, Garnica, Geisha and Caturra. In addition, these Arabica coffees are characterized by generating a greater economic return for producers [1].

On the other land, there are few studies regarding the use of by-products through local practices and traditional knowledge carried out by coffee-producing families. Coffee waste is often considered a problem, but it can be turned into value-added products and income diversification if managed with clean technologies and long-term waste management strategies. A first proposal that seeks to expand the usefulness of traditionally used by-products is to increase the knowledge of their organic and inorganic chemical composition. Several compounds, including lipids, proteins, lignin, pectin, cellulose, hemicelluloses and bioactive compounds such as antioxidants, caffeine, polyphenols, carotenoids, flavonoids and minerals, can be extracted through recycling, recovery or valorization in functional and traditional products [17]. Therefore, in this work, we also focus on the composition of soluble bioactive compounds in coffee by-products of origin in order to encourage their use in new products and possible recovery of bioactive compounds with potential agroindustrial use.

### 2.2. Soluble Bioactive Compounds in Coffee Cherry Cascara by HPLC-ESI-HRMS

The skin and pulp of the coffee cherry, popularly called cascara (husk), is the main underutilized by-product of coffee farming and is rich in macromolecules and bioactive compounds [4]. In the Sierra de Zongolica, 5% of the dried cascara is traditionally consumed as a tea-like infusion. These cascaras tea-like infusions are also consumed in other Latin American coffee-producing countries such as El Salvador, Nicaragua, Brazil and Bolivia [16], mainly for its pleasant taste, nutritional properties and the feeling of wellbeing it provides [15]. The dried and fresh cascara is also used for the production of spirits, soft drinks, concentrates and juices [4,11], as well as a commercial feed additive and animal feed [6,14].

Dried cascara is among the least-characterized coffee cherry by-products in terms of its composition of soluble and non-volatile bioactive molecules; recent studies focus mainly on its volatile molecules, odors and aromatic characteristics [15,16]. In this work, a metabolomic profile analysis based on HPLC-ESI-HRMS (Supplementary Figure S1), was conducted, and 93 non-volatile and water-soluble bioactive compounds were identified from a mixture of dried cascara Arabica (Arabica blend) and an individual sample of the dried cascara Garnica variety from the Sierra de Zongolica. These 93 detected molecules were classified into 13 groups of primary and secondary metabolites (Table 1), including organic acids (12), alkaloids (5), sugars (5), fatty acids (2), diglycerides (1), amino acids (18), phospholipids (7), vitamins (5), phenolic acids (11), flavonoids (8), chlorogenic acids (17), flavones (1) and terpenes (1). In contrast to the nonvolatile compounds in our study (Figure 1), Pua et al. [15] only report 51 soluble compounds, grouped into sugars (7), organic acids (6), methylxanthines or alkaloids (3) and polyphenols (35) detected and quantified by HPLC. Iriondo-DeHond et al. [16] report proteins, amino acids (18), lipids, fatty acids (23), sugars (5), fibers, minerals (8) and vitamin C from dried coffee cascara and from an instant powdered cascara beverage (freeze-dried).

The profile of polar and semi-polar metabolites varied in specific molecules among the thirteen chemical groups identified in the Zongolica dried cascara samples. The cascara Arabica (blend) had higher contents of organic acids, amino acids, cafestol, flavonoids, phenolic acids, vitamin B1 and minor alkaloids; in contrast, Garnica had higher contents of caffeine and chlorogenic acids (Table 1). In general, caffeine was the most abundant compound and alkaloid in both dried cascara samples, followed by quinic acid, chlorogenic acids, sugars such as glucose and sucrose, as well as lactic acid and amino acids such as valine and pipecolic acid, the flavonoid quercitrin and the flavanol catechin (Table 1). Other alkaloids identified were theophylline, theobromine and, in very low concentration, serotonin and trigonelline. Pua et al. [15] also identified the three most abundant alkaloids (methylxanthines) in our study.

Eighteen amino acids were identified in the Zongolica dried cascara, mainly essential protein amino acids (Table 1). Pipecolic acid is a non-protein cyclic amino acid like proline, is derived from L-lysine and was the second most abundant amino acid. This cyclic amino acid serves as a precursor for the biosynthesis of biological metabolites, so it has gained interest in recent years within the pharmaceutical and chemical industries [22]. Valine and 15 other essential amino acids were identified in the dried cascara, with the exception of cysteine, methionine, alanine and glycine. The chromatographic method does not allow the separation of the amino acids leucine and isoleucine; therefore, the value obtained is for both amino acids. Previously, Iriondo-DeHond et al. [16] reported 17 essential amino acids, with the exception of tryptophan, glutamate and aspartic acid, in dried Arabica coffee cascara and instant cascara powder (freeze-dried from aqueous cascara extraction). Other non-protein amino acids such as 4-hydroxyproline (Table 1) and aminobutyric acid (GABA) may also be present [23]. In our study, some amino acids were more than twice as high in the cascara of Arabica (blend), such as valine and arginine, and up to 20 times higher, such as tryptophan (Table 1). This metabolite is an essential amino acid, a key precursor for important molecules such as serotonin, melatonin and niacin, and it is used as a dietary supplement because of its many beneficial effects on human health [24].

Other primary metabolites identified were soluble sugars such as monosaccharides, disaccharides, trisaccharides and tetrasaccharides. Sucrose and glucose were the most abundant in both samples, followed by arabinose, the trisaccharide raffinose and the tetrasaccharide stachyose in Garnica (Table 1). Other monosaccharides reported in coffee cascara are mannose, fructose, galactose, xylose and maltose [15,23]. Sugars and amino acids play important roles in the Maillard reaction during drying of the cascara [25].

Lipids have important roles in the development of the coffee bean; they are precursors of the flavor, volatile compounds, aroma and body of the roasted coffee beverage. Lipids are also responsible for the effects of coffee on human health. Lipids represent about 7–17% of bean dry weight, with Arabica species containing the highest levels (15–17%) compared to Robusta (7–10%) [26]. In coffee, lipids include triacylglycerols (TGA, 75%), diterpenes (20%), sterols (5.5%), free fatty acids (1%), phospholipids (0.5%) and tocopherols (0.05%). Most of the fatty acids (free or esterified in TGA) are unsaturated, and the main ones are linoleic, oleic and linolenic acid. Lipids are mainly found in the endosperm as TGA, and only a small amount (2–3%) are present outside the seed [27]. There is little information on the lipid composition of coffee, mainly reported from green bean and roasted coffee [28,29]. In our study, we identified the saturated fatty acids arachidic and adipic, which are important in food (beverages), and the diglyceride (DG18:3,18:3); the diterpene cafestol, a common component of coffee beverages; tocochromanol, a lipophilic antioxidant of great importance for health; and seven phospholipids (Table 1) that can be sources of fatty acids [30]. Surprisingly, contrary to the higher concentration levels of bioactive molecules identified in the cascara Garnica, the phospholipid content was lower compared to the cascara Arabica.

Phospholipids are essential constituents of biological membranes, whose amphipathic molecule consists of a glycerol, two fatty acids, a phosphate group and a polar head group such as choline, ethanolamine, serine or inositol and are described as phosphatidylcholine (PC), phosphatidylethanolamine (PE), phosphatidylserine (PS) and phosphatidylinositol (PI), respectively [31]. In our results, the seven phospholipids identified were phosphatidylcholine (PC 16:0), unsaturated phosphatidylcholines (PC 18:1; PC 18:2), phosphatidylethanolamine (PE 18:0), phosphatidylinositol (PI 16:0) and phosphatidylserines (PS 17:1; PS 21:0). Silva et al. [28] report the same class of phospholipids in green coffee of Arabica species at different stage of fruit ripening and post-harvest processing; they are also present in roasted coffees and may have a relationship with coffee beverage quality [29]. Phospholipids can be present in some vegetables, fruits and juices [32] and, in our case, in coffee cascara.

Organic acids are the main non-volatile components responsible for the acidity of coffee [33]. The sensory characteristic, type and intensity of acidity presented by a food or beverage is dependent on the type, strength and concentration of the organic acid [16]. Twelve organic acids were identified in the mixture of Arabica and cascara Garnica, the most abundant being lactic acid, quinic acid, pyruvic acid, glutamic acid and shikimic acid. Citric, malic, citramalic and gluconic acids have also been reported in coffee cascara [34]. Pua et al. [15] report six organic acids in beverages prepared from cascara from wet processing of Arabica coffees in Brazil, Guatemala, El Salvador and Papua New Guinea. The organic acids reported were citric, tartaric, malic, succinic, fumaric and gallic; the content of individual organic acids varied among the blends of Arabica cascara from different countries. The Papua New Guinea blend had the highest level of total organic acid content, and the Arabica blend from El Salvador the lowest [15]. In our results, the cascara Arabica presented higher levels of organic acids than the cascara Garnica (Table 1).

The presence of lactic acid in the dried cascara is possibly due to natural coffee cherry fermentation and the drying of the cascara. In fact, by-products of fruits, vegetables and cereals, being rich in nutrients such as sugars, are fermented by lactic acid bacteria. In many cases, this fermentation degrades macromolecules and toxic substances, facilitates the availability and absorption of nutrients, masks off-flavors or creates new flavors and odors of interest in foods [35]. Fermentation of some plant by-products with lactic acid bacteria can produce livestock feed, novel foods, functional fruit drinks and fruit by-products [35,36], as well as fermented tea leaves to improve health [37,38]. Fermentation develops intense flavor and aroma in coffee beans for the specialty market and likewise in dried and fermented coffee cascara. DePaula et al. [23] report more complex, exotic and intense sensory properties, odor and flavor in fermented than non-fermented cascara. In specialty coffees, citric, malic, tartaric and succinic acids may be present in coffee beans during wet fermentation. Martinez et al. [39] mention that some organic acids in coffee are of natural origin through metabolic reactions within the fruit, others originate from prefermentations that occur after the cherry harvest, and others can develop or increase during the wet fermentation process by bacteria and yeasts and by beneficial microorganisms in controlled fermentation.

Quinic acid was the most abundant organic acid in the cascara Arabica and cascara Garnica, but less abundant in the Mexican variety. Quinic acid is an antioxidant that contributes to the flavor and taste of plant-derived foods [40]. In fruits, quinic acid is found in free form along with other organic acids such as tartaric, malic, citric, succinic and shikimic acids, which can act together as inhibitors of oral pathogens that cause caries and gingivitis [41]. In addition, quinic acid shows several therapeutic properties as an antimicrobial, antifungal, antibiofilm, cytotoxic, antidiabetic, insecticidal, anticancer, antioxidant and analgesic agent [42]. Quinic acid can also be bound to hydroxycinnamic acid residues, forming esters called chlorogenic acids [40].

In aqueous extracts of dried cascara, the total polyphenol content varies between 4.9 and 9.2 mg gallic acid equivalents (GAE)/g, with an antioxidant activity between 51 and 92 µmol Trolox equivalents (TE)/g, depending on the coffee variety [11]. The polyphenols identified in cascara Arabica (blend) and cascara Garnica were 38 and were distributed among flavonoids and flavonols (9), phenolic acids (11), chlorogenic acids (17) and caffeine. In addition to catechin (flavonol), mainly in the Mexican variety, the eight flavonoids are represented by hyperoside, isogentisin, kaempferol, naringin, quercetin, quercetin-3-O-glucoside, rutin and quercitrin, the latter being the most abundant of the flavonoids. In polyphenolic fractions of dried pulp, six flavonoids have been reported, including quercetin, naringin and catechin, in addition to five phenolic acids and caffeine [43].

In our data, phenolic acids included hydroxycinnamic acids and their derivatives, including cinnamic, caffeic, ferulic, coumaric and synaptic acids, and hydroxybenzoyl acids represented by vanillic, gallic and syringic acids. Chlorogenic acids were the most abundant among all polyphenols. Seventeen chlorogenic acids or quinic acid esters with hydroxycinnamic acids such as caffeic acid, ferulic acid, cinnamic acid and coumaric acid were identified in our cascara samples. Chlorogenic acids with caffeic acid were the most representative, with 4-O-Caffeoylquinic acid being the most abundant. Like quinic acid, chlorogenic acids together with caffeine have antimicrobial [20], antioxidant and anti-inflammatory properties [44].

Vitamins are necessary and essential, in small amounts, for the proper functioning of the metabolism of a living organism; therefore, they must be provided through food intake [45]. Products of plant origin, including fruits and vegetables, are natural sources of vitamins, although some may be at low concentrations, such as B complex. Vitamins are important antioxidants, essential compounds and are indispensable for optimal health [46]. Nicotinic acid (vitamin B3), thiamine (vitamin B1), pyridoxine (vitamin B6), riboflavin (vitamin B2) and alpha-tocopherol (vitamin E) were identified in both cascara samples (Table 1). Water-soluble B-complex vitamins were the most abundant, mainly vitamin B3, and vitamin B1 was twice as abundant in the Arabica blend. Vitamin E has been identified in silverskin and may have potential uses in food, cosmetics and pharmacology [12]. Vitamins B2 and B3 have also been identified and reported in silverskin [47]. In coffee cherry pulp juice concentrate, there are vitamin E [48] and nicotinic acid [6]; moreover, Iriondo-DeHond et al. [23] report vitamin C in instant pulp beverages. B vitamins and vitamin C are common in the aqueous part of fruits, foods and beverages. Vitamin E is common as the lipid fraction in prepared roasted coffee beverages [28]. In addition, vitamins B2 (0.18–0.2 mg/kg) and B3 (2.5–3.1 mg/kg) were studied and reported for the first time in silverskin [47].

### 2.3. Rapid Analysis of Caffeine and Other Compounds in Coffee By-Products by DART-MS

The composition of bioactive molecules smaller than 1000 Da was explored using high-resolution ambient DART-MS time-of-flight (TOF) mass spectrometry. DART-MS has been used for chemical fingerprinting, metabolomic profiling and clustering of small plant molecules due to its rapid, direct measurement in real time, without preparation or separation, of plant samples or extracts [49,50]. In roasted coffee, DART-MS has been used for caffeine analysis of different roasted coffee beverages made from Arabica and Robusta species [51], but there are no reports of analysis in coffee by-products. In contrast to the husk (cascara) from wet processing [15,34], the chemical composition of by-products such as defective and healthy coffee beans, mucilage, parchment [13] and unroasted silverskin are still poorly explored.

For DART-MS analysis, extractions were performed on the dried cascara, parchment, unroasted silverskin and green coffee of the Arabica blend, using water as a solvent at temperatures from 92 to 121 °C for 10–20 min. Water is the safest non-toxic, environmentally friendly universal solvent for the production of food and non-toxic extracts. Aqueous extraction, with its temperature, solid-liquid ratio (*w*/*v*), particle size and extraction time, allows the obtaining of extracts that are rich in bioactive compounds such as phenolic compounds and caffeine, which is similar to the use of solvents or alternative chemical and physicochemical methods [18]. The best conditions reported for the recovery of bioactive compounds in dried cascara are a solid-liquid ratio of 1–10 *w/v* and 1–100 *w*/*v*, a temperature of 85–100 °C for 10 to 15 min and a particle size of 1.4 mm of the by-product [18].

The dried cascara is traditionally consumed as a tea-like infusion in producing countries, due to its pleasant flavor, aroma and nutritional properties [18], and it has been authorized by the European Food Safety Authority (EFSA) as a novel food for commercialization in the European Union [52]. For this reason, the extraction of bioactive compounds from the dried cascara in our study was similar to the domestic process of preparing tea-like infusions using boiling water (92 °C) for ten minutes. Parchment, silverskin and green coffee, being resistant materials rich in dietary fiber and lignin, were autoclaved for 20 min at 121 °C. The solid–liquid ratio was 1/100 *w/v* for silverskin and 1/10 *w/v* for the other by-products. Silverskin is a solid material with a large volume and absorbs a large amount of liquid; therefore, extraction with a different *w/v* ratio was necessary.

DART-MS analysis of dried cascara, parchment, silverskin and green coffee is presented in Table 2 and Supplementary Figure S2. The table shows the metabolites with molecular form M that were assigned to the precursor ions or molecular adducts observed in the DART spectra of the coffee by-products. Several molecular ions (*m*/*z*) were observed in both positive and negative modes, and the *m*/*z* ion intensity varied among by-products, indicating the abundance of the bioactive compounds in each sample by relative intensity (R.I. %). The majority of the adducts (*m*/*z*) are attributed to caffeine, chlorogenic acids, sugars, polyphenols and flavonoids. In all samples, the most abundant ion (100% RI) in positive mode was 195.16 *m*/*z* in dried cascara and 195.08 *m*/*z* in the other by-products, corresponding to the adduct type [M + H]^+^ and representing caffeine. Other important compounds were caffeic acid and quinic acid. The adducts [M + H]^+^ were observed as 181.14 *m*/*z* and 181.08 *m*/*z* for caffeic acid and 193.13 *m*/*z* and 193.08 for quinic acid, and correspond to dried cascara and parchment coffee by-products, respectively. The presence of phenolic acid and quinic acid may be related to the main chlorogenic acid observed in dried cascara, 4-O-Caffeoylquinic acid (Table 1). However, in DART positive mode, very low signals of the [M + H]^+^ adduct of chlorogenic acids were observed (Table 2), but there are reported signals of the fragmentation of the isomers of the compound [53].

Wang et al. [54], using soluble monosaccharide standards, reported the identification of saccharides by DART-MS in positive mode. The glucose adducts observed in the positive mode were 198, 180, 163 and 127 *m*/*z* (Table 2). The molecular ion 198 *m*/*z* corresponds to the main adduct [M + NH_4_]^+^; this is a signal of six-carbon sugars or an ammoniacal fragment of monosaccharides [54]. In the extracts of by-products and green coffee, the presence of the adduct [M + NH_4_]^+^ was observed, and the relative intensity (%) of the ammoniacal molecular ion, in decreasing order, was parchment, silverskin, dried cascara and green coffee (Table 2). Other molecular ion signals in positive mode were observed in the parchment extract. 303.12 *m*/*z* seems to correspond to quercetin in the form of adduct [M + H]^+^ as reported [56]. Quercetin was one of the flavonoids identified in the dried cascara (Table 1) and has been reported in roasted silverskin [57].

The dried cascara infusion and the extracts of parchment, silverskin and green coffee in negative mode showed signals of caffeine, chlorogenic acids and sugars as the most abundant metabolites. In negative mode, the adduct [M-CH_3_]^−^ was observed, corresponding to 179.06 *m*/*z* and 179.05 *m*/*z* of caffeine in cascara and by-products, respectively. This adduct was more abundant in parchment and green coffee. Chlorogenic acids were observed as 191.06 *m*/*z* and 191.05 *m*/*z* in the by-products (Table 2). Previous reports in negative mode MS/MS indicate the presence of molecular ions 353, 191, 179 and 173 *m*/*z* for the identification of caffeoylquinic acid (CQA) isomers using standards. The 191 *m*/*z* ion is the main product of CQA fragmentation, followed by 179 *m*/*z* and 173 *m*/*z*; the presence or absence of these ions is used for the identification of 5-CAQ, 4-CQA and 3-CQA isomers [53]. In our DART-MS analysis, the abundance of the three main CQA fragments was observed mainly in green coffee, which would indicate the presence of 4-CQA. Likewise, the presence of the molecular ion 533.18 *m*/*z* was observed, which could correspond to an adduct type [M + OH]^−^, as indicated by Cody et al. [49], and may be related to chlorogenic acids of the dicaffeoylquinic acid type. The molecular ion 371.12 *m*/*z* coincides with the fragment of this type of chlorogenic acid and is present in all by-products, mainly in green coffee and parchment. This molecular ion could be the result of the loss of 162 *m*/*z* of the -C_9_H_6_O_3_ fragment of the [M + OH]^−^ adduct suggested for dicaffeoylquinic acid. Other fragments related to dicaffeoylquinic acid are 353 *m*/*z* and 335 *m*/*z* [58], which could be the result of -H_2_O desorption during ambient ionization.

The molecular ion 215 *m*/*z* is of the [M + Cl]^−^ type and is related to six-carbon sugars such as glucose, and the molecular ion 359.12 *m*/*z* to disaccharides [55]. The [M + Cl]^−^ adduct was less abundant in green coffee but between 100–80% relative intensity in the other by-products, mainly in silverskin and parchment (Table 2). The ion 179 *m*/*z* is related to hexoses and caffeine in negative mode [34]. Its presence could be associated with the abundance of both molecules, but in DART-MS, the formation of the 215 *m*/*z* adduct for hexoses seems to be favored [55]. Molecular ions of pentose-related adducts, pectin and mucilage are observed as reported by Procacci et al. [55]. Sugars are major compounds in fresh pulp, which is also a source of pectin as a food ingredient [18]. Although parchment, silverskin and green coffee are composed of insoluble carbohydrates and dietary fiber [17], during pulping (wet methods), the sugars from the mucilage on the parchment can be diffused into the silverskin and green bean, hence its abundance in DART-MS analysis. Silverskin is rich in glucose, as well as other monosaccharides such as fructose, xylose, arabinose, mannose and galactose [12].

Dried cascara teas can have different aroma and flavor attributes, similar to honey, nuts, herbal, woody, black teas, citrus and fruit, that can vary depending on whether they were fermented or not [15,16]. Like specialty coffees, pulp fermentation favors other flavors and odors demanded by sophisticated markets and consumers [16]. From our data, tea-like infusions of dried cascara seem to be important sources of different bioactive compounds, caffeine, chlorogenic acids and sugars (Table 1 and Table 2).

Few studies have reported the metabolomic profile of parchment [59], although its valorization potential is significant as a source of caffeine and chlorogenic acids [19], which can be used as antioxidants and antimicrobials. In the case of silverskin, only the characterization of the roasted waste is reported [60,61], with the presence of lipids and melanoidins due to the roasted process, in addition to caffeine and chlorogenic acids such as 3-CQA and 4-CQA [19]. In green coffee, previous studies report amino acids, polyamides, caffeine and antioxidant compounds [21,62]. In our spectra, caffeine, chlorogenic acids and sugars were mainly observed in these three by-products, varying in relative intensity and signals of chlorogenic acids with one or two caffeic acid molecules.

### 2.4. Mineral Content of Green Coffee and By-Products of Primary Coffee Processing

**Figure 3:**
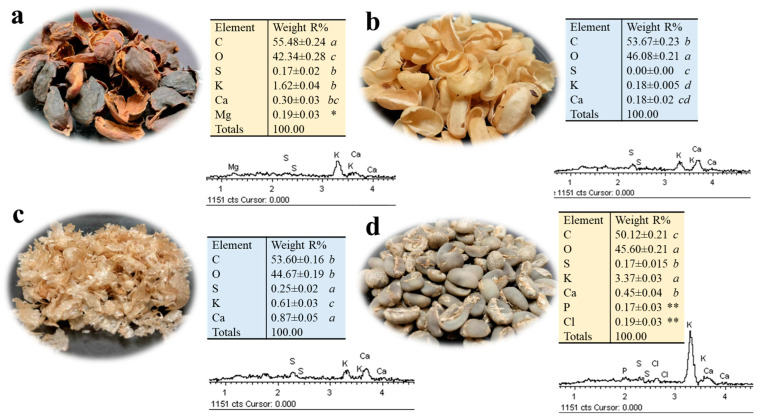
Mineral microanalysis in cascara, parchment, silverskin and green coffee by energy dispersive X-rays spectroscopy (EDS). (**a**) Cascara (husk), (**b**) parchment, (**c**) silverskin and (**d**) green coffee. The relative content of each element is indicated as a percentage. The spectra show the identification of each chemical element by its characteristic X-ray lines (KeV). Five measurements were made per coffee by-product and green coffee sample. Data are reported as mean ± standard error of the mean (*n* = 5). Different letters (italics) indicate significant differences between samples for element (*p* < 0.001; Tukey’s test). Asterisks indicate the presence of the element in the sample, * magnesium (Mg) and ** phosphorus (P) and chlorine (Cl).

In coffee, the value of ash from by-products of origin is high (3–10%), which is attributed to mineral elements. The mineral content represented as ash was reported as 6–10% in dry cascara (pulp and skin), 3–7% in husk (dry process), 0.43% in mucilage [23], 3–5% in green coffee [4], 1% in parchment, 5–7% in roasted silverskin [19] and >1% in spent coffee [63]. The mineral composition of the by-products of primary coffee processing (dried cascara, parchment, silverskin and green coffee) from our analysis is presented in Figure 3. The organic composition was mainly represented by 42–46% oxygen (O) and 49–55% carbon (C), depending on the by-product. The macronutrients were potassium (K), phosphorus (P), calcium (Ca), magnesium (Mg) and sulfur (S), and the micronutrient was chlorine (Cl). These mineral elements were significantly different in their presence and relative element content (%) among by-products (Figure 3).

In dried cascara, the K content was 1.62% and, at a lower level, Ca (0.30%), Mg (0.19%) and S (0.16%) (Figure 3). Machado et al. [8] mention K, Ca and Mg as macronutrients found in coffee cascara and silverskin. Together with organic bioactive compounds, mineral composition adds nutritional value [17] and improves the physicochemical properties of foods made from coffee cherry cascara such as jams, juices, concentrates, beverages and gelatin. Coffee cascara flour is used for breads, cookies, muffins, squares, brownies, pastas and sauces [4]. For example, Ca and Mg, in synergy with pectin, are essential for the gelatinization of jams. Also, acidic foods increase the solubility of minerals and improve absorption [17].

Coffee by-products contain key elements needed by plants to complete their life cycles, including K, Mg, Ca and other trace elements [64]. Dried cascara (40%) is used in Zongolica as a fertilizer by direct application to the soil around plants, so that the plants can use the macronutrients present (Figure 3), mainly K. Kasongo et al. [65] report that the application of cascara and pulp increased the biomass of a forage crop, with a good absorption of macronutrients (Ca, Mg, K, N and P), but with a decrease in the amount of micronutrients. In coffee-producing countries such as Costa Rica, the CoopeTarrazu cooperative composts the cascara with other fertilizers that enrich the mineral content to obtain an organic fertilizer (EcoFértil) applied to coffee crops. Wastes such as spent coffee grounds have been used as biochelators for the biofortification of edible plants and as organic fertilizer in lettuce crops, as an ecological alternative to inorganic fertilizers (NPK), producing lettuces with a higher content of essential elements and greater nutritional value [66,67]. Recently, the application of spent coffee ground infusions was shown to improve growth and photosynthesis in cucumbers, but the polyphenolic composition can have negative effects on plants [68].

Coffee by-products used as organic compost and biofertilizers can return organic and inorganic matter and improve biological fertility, soil structure and water retention capacity, but there are some concerns about high humidity, acidic pH and polyphenolic compounds, such as melanoidins and caffeine, that may or may not favor the soil [66,67]. For example, the use of spent coffee as compost stimulates microbial growth and nutrient competition with plants. Polyphenols and caffeine can be phytotoxic but can be removed with vermicomposting and pyrolysis (400 °C), although it is not recommended to remove all organic compounds from by-products due to their properties as biochelates [13]. Schmidt Rivera et al. [69] mention that the direct application of by-products on agricultural lands, together with their incineration, is one of the most sustainable and least environmentally impactful ways of using coffee waste and by-products. In the valuation of spent coffee, biodiesel production is the least sustainable option, followed by composting [69].

Parchment and silverskin (unroasted) present the same macronutrients, but silverskin has the highest content of Ca (0.86%), K (0.60%) and S (0.25%) (Figure 3). Nzekoue et al. [47], in a mineral analysis of silverskin obtained from roasted coffee, report high contents of Ca (1.1%), K (1%), micronutrients such as iron (Fe) (0.24%), and trace concentrations of elements such as manganese (Mn), copper (Cu) and zinc (Zn). The difference in macronutrient content between roasted silverskin and our silverskin samples was possibly due to the loss of volatile compounds and water during roasting. Some current applications of roasted silverskin and spent coffee are as substrates for fungal growth or for composting and vermicomposting [70].

The K (3.36%), Ca (0.44%), Cl (0.19%), P (0.17%) and S (0.17%) represent the mineral content of green coffee in decreasing order (Figure 3). Green coffee is a rich source of K [71], an element that is important for its multiple biological functions. Granulated or broken green coffee is used as a food supplement and tea-type drinks called “white coffee” and are a source of caffeine, chlorogenic acids and minerals, which are reported between 3–5% [4,21]. The acceptance of green coffee drinks and roasted coffee can be related to personal preference, to the caffeine content for its stimulating properties and to the content of chlorogenic acids for their antioxidant activity [72]. In contrast to the macronutrients and micronutrients in green coffee, significant contents of aluminum (Al), chromium (Cr), cadmium (Cd) and lead (Pb) can be found [12,71], which could be harmful to health if solubilized. However, due to the agroecological characteristics and local practices of the Sierra de Zongolica, the sale of green coffee as a food supplement and antioxidant tea-type drinks should not be a problem.

## 3. Materials and Methods

### 3.1. Ethnographic Study Area

The study was carried out in the coffee-growing region of the Sierra de Zongolica, Veracruz. In 2010, this area received a designation of origin for coffee from the Mexican Institute of Industrial Property (IMPI). This region is divided into a temperate and a warm zone. Due to the agro-environmental characteristics, Arabica coffee varieties (*Coffea arabica*) such as Typica, Bourbon and Caturra are grown, as well as Robusta coffee varieties (*Coffea canephora*); hybrid varieties of Arabica coffees such as Garnica, Geisha and Ciruela Hidalgo; and hydrid varieties of Arabica and Robusta known as Catimores (Costa Rica 95, Colombiano, Salchimor, Oro Azteca and Anacafé) [73]. A detailed qualitative ethnographic investigation was carried out using direct and participatory observation, semi-structured interviews, surveys, life stories, field diaries and plot-level tours, mainly observing the local practices of producers regarding by-products and waste from wet and dry coffee processing. During the field study in October–November 2021, sampling of by-products of coffee cherries that are reused in a traditional way in Zongolica was carried out for chemical characterization in the laboratory.

### 3.2. Plant Material

The by-products and green coffee were obtained from the wet processing of Arabica coffee cherries by families of the Nahuatl population of Tlecuaxco, in the Sierra de Zongolica, Veracruz, Mexico. The wet processing of the cherries, known as “washed coffees”, was according to the local practices and knowledge of the Tlecuaxco community known as [74]. The coffee cherry harvest was carried out in the morning and consisted of the manual collection and selection of ripe (red) fruits of Arabica species (Costa Rica 95 and Garnica). The coffee cherries were washed with water, and the empty fruits were removed; then, the fruits were passed through a pulping machine (electric) that removed the pulp (mesocarp) and outer skin (exocarp) of the beans. After pulping, the beans were washed and fermented for 24 h to remove the mucilage (internal mesocarp). The fermented beans were washed again and dried for 10 days on wooden sieves with wire mesh to obtain parchment coffee (Figure 2). Parchment coffee is a form of long-term commercialization and storage of coffee beans (Figure 2) that consists of the bean or green coffee (endosperm and embryo), silverskin (testa or epidermis) and parchment (endocarp). Likewise, the pulping by-product (mesocarp plus exocarp) was dried in the air on wire mesh sieves for 4 days. This dried by-product composed of pulp and outer skin was considered as cascara (the Spanish name for husk) [11]. For our analysis, a pool of dried cascara (1 kg) and a pool of parchment coffee (1 kg) from the blend of Arabica coffees were sampled and stored in polypropylene bags until processing, extraction and determination of bioactive compounds in the laboratory.

Additionally, a pool of fresh cherries from a blend of Arabica coffees (Costa Rica 95 and Garnica) and a pool of the Mexican Garnica variety were used. The cherries were kept fresh until processing after 24 h of harvesting and manual selection. Damián Xotlanaihua-Flores, a native of Tlecuaxco, provided all samples of dried plant material (dried cascara and parchment coffee) and fresh coffee cherries.

### 3.3. Sample Preparation

Coffee cherries harvested in the Sierra de Zongolica (Veracruz), which are representative of Arabica varieties (blend) and the Garnica variety, were washed three times with tap water and twice with distilled water. Subsequently, the peel (skin and pulp) was manually separated from the fruits; these were placed in glass Petri dishes and dried in a convection oven at 60 °C for 72 h. Both dried peel (cascara) samples were ground separately in an industrial blender (Oster, Mexico) and subsequently in a Retsch MM400 micromill with Zirconium beads until obtaining a fine and homogeneous powder. Each dried cascara sample was recovered and stored in polyethylene plastic bags until its metabolomic analysis by High Pressure Liquid Chromatography coupled to High-Resolution Mass Spectrometry (HPLC-ESI(+/−)-HRMS).

On the other hand, the parchment coffee obtained from the wet processing of coffee cherries from the Arabica blend (Figure 2) was manually separated into parchment, silverskin and green coffee; these samples were stored in polypropylene bags. Subsequently, a pool of 50 g of samples from the wet processing of coffee (dried cascara, parchment, silverskin and green coffee) were ground separately in an industrial blender (Oster, Mexico) until obtaining a particle size of less than 1.5 mm (mm). The samples with millimeter particle sizes were used for aqueous extractions and qualitative analysis of the chemical profile by Direct Analysis Real-Time Mass Spectrometry (DART-MS).

A 10 g pool of by-products and green coffee from the wet process with millimetric particle size were ground separately in a Retsch MM400 zirconium bead micromill (Retsch GmbH, Haan, Germany) at a frequency of 22.0 1/s for 9 min. The microground material obtained from each sample was stored in polyethylene bags for multielement microanalysis by energy-dispersive X-ray spectroscopy coupled with scanning electron microscopy (SEM-EDS).

### 3.4. Extraction and Analysis of Polar and Semi-Polar Metabolome of Cascara by HPLC-ESI (+/−)-HRMS

Targeted analysis of polar and semi polar metabolites was performed as reported by Carcea et al. [73] with slight modifications: 10 mg of dried cascara powder were extracted with 750 µL of 75% (*v*/*v*) cold methanol with 0.1% formic acid (Sigma-Aldrich, Cat. 5630-50ML-F), spiked with 0.5 mg/mL formononetin (Sigma–Aldrich, Cat. No. 47752-25MG-F). High Pressure Liquid Chromatography coupled to High-Resolution Mass Spectrometry (HPLC-HRMS) analysis was carried out as previously reported [75] by using HPLC-HESI-HRMS (Ultimate3000 Dionex/Q-Exactive Orbitrap, Thermo Fisher Scientific, Waltham, MA, USA), operating in both negative and positive ion modes (Supplementary Figure S1). Targeted metabolite identification was performed through the comparison with authentic standards, when available, and by interrogation of a custom database and the Metlin database [76] on the basis of the accurate masses obtained from the Pubchem database (http://pubchem.ncbi.nlm.nih.gov/, accessed on 19 May 2024) for native compounds or from the Metabolomics Fiehn Lab Mass Spectrometry Adduct Calculator (http://fiehnlab.ucdavis.edu/staff/kind/Metabolomics/MS-Adduct-Calculator/, accessed on 19 May 2024) for adducts. Quantification was performed as fold internal standard, formononetin.

### 3.5. Aqueous Extraction of Coffee By-Products and Green Coffee

Preliminary experiments in the laboratory indicated that the particle size influenced the yield of bioactives extracted from dried cascara and green coffee, and millimetric granulometry (<1.5 mm) was the best option for the aqueous extraction of compounds and antioxidant activity. Therefore, all dried samples (Arabica blend) of by-products and green coffee for aqueous extractions had particle sizes less than 1.5 mm (Section 3.3). For the preparation of aqueous extractions, the conditions reported by Hu et al. [18] were followed.

The dried coffee cascara sample (1 g, Arabica blend) was extracted with 10 mL of distilled water at a 1:10 (*w*/*v*) ratio for 10 min at 92 °C [11,18]. The aqueous extraction was filtered with a 0.45 µm PVDF membrane (Millex-HV) and then stored at −20 °C in Eppendorf tubes prior to DART-MS analysis. For parchment and green coffee, a 1:10 (*w*/*v*) ratio was used, 10 g were weighed per sample, and 100 mL of distilled water was added. For silverskin, due to the volume of the material, a sample ratio of 1:100 (*w*/*v*) was used, 5 g were weighed, and 500 mL of distilled water was added. Solid–liquid (S-L) extraction of parchment, silverskin and green coffee was completed in an autoclave at 121 °C and 1.5 atm for 20 min. The aqueous extractions were filtered through a 0.45 μm PVDF membrane (Millex-HV).

All aqueous extracts of coffee by-products and green coffee were stored in Falcon tubes and Eppendorf tubes at −40 °C until analysis by DART mass spectrometry. The extracts were performed in duplicate.

### 3.6. DART Mass Spectrometry of Aqueous Extractions of By-Products and Green Coffee

DART mass analysis was carried out on a JMS-T100LP AccuTOF LC-PLUS spectrometer (JEOL, Tokyo, Japan) with a DART SVP100 ion source, (Ionsense, Saugus, MA, USA). The DART ion source was operated with helium for analysis and nitrogen for standard mode, the gas temperature was 300 °C, the inlet pressure was 0.55 MPa, and the voltage was ±600 V in standard mode positive and negative ions. The instrument was calibrated with a solution of PEG-600 in methanol. MS-DART analysis was performed by immersing a borosilicate capillary tube in the solution of the by-products or green coffee aqueous extract; subsequently, the capillary tube was located ≥1 cm between the helium stream of the DART ionization source and the vacuum interface for ionization and subsequent determination of *m*/*z* molecular ions in the AccuTOF detector. The acquisition of mass spectra was recorded with Mass Center System Software Version 1.5.0k, setting a mass range of *m*/*z* 50–1000 Daltons (Da). Each sample was detected at least three to five times for 2–5 min. In addition to *m*/*z* spectra, data on molecular ions *m*/*z*, intensity and relative abundance were obtained with Mass Center System Version 1.5.0k software. For the identification of metabolites in coffee samples, a comparison was made with monosaccharide [54,55], caffeine and chlorogenic acid standards. Standards were examined in positive and negative mode, under the same conditions used for the aqueous extracts. All standards were purchased from Sigma Aldrich (China and Italy) and were prepared at 1 mg/mL in HPLC water (Sigma Aldrich, USA).

### 3.7. SEM-EDS Mineral Microanalysis Method

By-products and green coffee samples from the wet coffee process were ground in a zirconium bead micromill as described in Section 3.3. Determination of the relative mineral content was performed with a scanning electron microscope (SEM) (JEOL, Japan; model JSM6390LV/LGS; INCA^®^ Suite 4.08 software) equipped with an energy-dispersive electron probe X-ray (EDX) system (LK-IE250 Oxford INCA Energy 250) for qualitative and quantitative microanalysis [55]. Briefly, 2–2.5 mg of micro-milled powder from each sample was weighed; each powder was carefully placed on a metal stud using double-sided carbon tape. The EDS (EDX) scan time was set to 70 s, in a 3.01 × 2.32 mm area or spot ROI with a magnification of 40X (SEM) and a scan energy of 20 Kv [22]. At least five EDS determinations were performed per sample. The data were processed by descriptive statistical analysis, mean and standard deviation.

### 3.8. Statistical Analysis

To determine significant differences between the compounds of the cascara Arabica and the cascara Garnica, the Student’s *t*-test was applied (Excel Office 365). The EDS microanalysis was evaluated by one-way variance analysis (Sigma Plot ver. 12.0, Software). The means of each element per sample (by-products and green coffee) were compared with Tukey’s multiple range test.

## 4. Conclusions

Metabolomics and mineral profiling can enhance the link between the quality of green coffee and primary coffee by-products with the traditional cultivation area and its local practices. The objective of our work was to identify organic and inorganic bioactive compounds of green coffee and coffee by-products related to the production of origin in the Sierra de Zongolica, as well as the roles these compounds can play in the valorization and reuse of by-products and the trade of green coffee.

A first metabolomics analysis by HPLC-ESI(+/−)-HRMS of the aqueous extracts of dried cascara, Arabica blend and Garnica variety, identified 93 polar and semipolar nonvolatile molecules belonging to 13 chemical groups: organic acids, alkaloids, sugars, fatty acids, amino acids, diglycerides, phospholipids, vitamins, phenolic acids, flavonoids, chlorogenic acids, flavones and terpenes. The dried Cascara of the Arabica blend highlighted organic acids, alkaloids (theobromine and theophylline), amino acids (tryptophan and valine), diterpenes, flavonoids and phenolic acids. In contrast, the dried cascara of Garnica highlighted caffeine, chlorogenic acids, phospholipids and sugars.

A rapid qualitative analysis by DART-MS revealed the main components of the profile of aqueous extracts of dried cascara, parchment, silverskin (unroasted) and green coffee. The most abundant components were caffeine, chlorogenic acids, sugars, polyphenols and flavonoids. The aqueous extract of green coffee was abundant in caffeine and chlorogenic acids. The dried cascara infusion showed the presence of caffeine, chlorogenic acids, sugars and pectin-related residues. In silverskin (unroasted) and parchment, the presence of caffeine, chlorogenic acids and sugars was observed; their extracts can be used as antioxidants, natural herbicides and antimicrobials.

Mineral microanalysis by SEM-EDS of by-products and green coffee revealed the presence of five macronutrients (P, Ca, P, Mg, S) and one micronutrient (Cl). Potassium (K) was the most abundant mineral, mainly in green coffee and dried cascara, followed by silverskin and parchment, while Ca was twice as high in silverskin. In general, green coffee presented the highest mineral abundance, followed by dried cascara with the presence of Mg.

The bioactive compounds from these by-products and green coffee can be used and recycled in the production of beverages, dairy and bakery products as antioxidants and colorants, as well as in the pharmaceutical industry; they have applications in anti-aging and anti-wrinkle products and as protective agents in different cosmetics. They can also be used as fertilizers, composts and herbicides for weed control in agroecology, and as antimicrobials in construction materials such as wood and stone.

On the other hand, the composition of bioactive molecules in green coffee and by-products can vary significantly depending on the processing method, the species and variety of coffee and the type and origin of by-product. In addition, the roasting and preparation of the beverage influences the waste generated in the places of consumption. We will take this perspective into account for future advances in our study.

Finally, we believe that the integration of local coffee-growing practices and knowledge with the reuse of by-products, scientific knowledge and available technological innovation can generate a synergy with positive effects at the economic, environmental and social levels in the coffee-growing communities of the Sierra de Zongolica, where the environmental and economic crises of the global coffee market have led to the prevalence of conventional traders.

## Figures and Tables

**Table 1:** Polar and semi-polar compounds in cascara Arabica (A) and cascara Garnica (B) samples quantified by HPLC-ESI (+/−)-HRMS. Data are reported as relative to the internal standard (see Materials and Methods for details).

Metabolite Class	Cascara Arabica (A)	Cascara Garnica (B)	AVG A/B	*p* Value A/B
**Organic acids**				
*Citraconic acid*	0.16 ± 0.03	0.10 ± 0.02	**1.61**	0.0113
*Glutaric acid*	9.88 ± 1.01	1.91 ± 0.23	**5.18**	0
*Glycolic acid*	0.51 ± 0.04	0.47 ± 0.06	1.09	0.2916
*Itaconic acid*	0.39 ± 0.09	0.43 ± 0.04	0.92	0.521
*Lactic acid*	125.77 ± 7.31	174.81 ± 8.81	**0.72**	0.0001
*Malic acid*	0.21 ± 0.06	0.13 ± 0.08	1.67	0.1461
*Oxalic acid*	0.04 ± 0.04	0.18 ± 0.06	**0.21**	0.0068
*Pyruvic acid*	15.73 ± 0.71	18.63 ± 2.97	0.84	0.1068
*Quinic acid*	1001.62 ± 97.4	799.13 ± 54.55	**1.25**	0.011
*Shikimic acid*	2.71 ± 0.22	0.90 ± 0.13	**3**	0
*Succinic acid*	1.85 ± 0.26	1.16 ± 0.14	**1.6**	0.0032
*Tartaric acid*	1.84 ± 0.29	0.37 ± 0.15	**4.99**	0.0001
**Alkaloids**				
*Caffeine*	4637.42 ± 248.28	5084.88 ± 151.08	**0.91**	0.0217
*Serotonin*	0.04 ± 0.02	0.25 ± 0.05	**0.15**	0.0004
*Theobromine*	9.50 ± 1.05	7.43 ± 0.52	**1.28**	0.0126
*Theophylline*	10.25 ± 0.66	2.41 ± 0.14	**4.25**	0
*Trigonelline*	0.01 ± 0.00	0.01 ± 0.01	0.54	0.2815
**Amino acids**				
*4-Hydroxyproline*	0.85 ± 0.13	0.91 ± 0.12	0.93	0.5246
*Arginine*	0.38 ± 0.10	0.16 ± 0.06	**2.43**	0.0072
*Asparagine*	12.86 ± 0.92	6.96 ± 0.15	**1.85**	0
*Aspartic acid*	6.92 ± 0.66	14.50 ± 3.14	**0.48**	0.0033
*Glutamic acid*	4.18 ± 2.30	5.25 ± 0.42	0.8	0.3964
*Glutamine*	0.42 ± 0.03	0.36 ± 0.03	**1.17**	0.0376
*Histidine*	0.07 ± 0.04	0.12 ± 0.01	**0.55**	0.0306
*Leucine-Isoleucine*	1.50 ± 0.07	2.95 ± 0.40	**0.51**	0.0004
*Lysine*	0.12 ± 0.06	0.22 ± 0.06	**0.52**	0.0376
*Phenylalanine*	4.50 ± 0.32	7.31 ± 0.78	**0.62**	0.0006
*Pipecolic acid*	27.43 ± 0.82	27.04 ± 4.92	1.01	0.8808
*Proline*	8.96 ± 12.79	17.82 ± 11.39	0.5	0.341
*Serine*	0.02 ± 0.01	0.02 ± 0.01	0.88	0.7755
*Threonine*	0.11 ± 0.01	0.10 ± 0.02	1.1	0.4729
*Tryptophan*	2.25 ± 0.66	0.11 ± 0.02	**21.26**	0.0006
*Tyrosine*	4.55 ± 0.36	3.35 ± 0.44	**1.36**	0.0057
*Valine*	37.86 ± 6.90	13.63 ± 2.01	**2.78**	0.0005
**Fatty acids**				
*Adipic acid*	0.72 ± 0.29	0.62 ± 0.25	1.16	0.6113
*Arachidic acid*	23.05 ± 1.17	24.05 ± 2.57	0.96	0.5063
***Diglyceride***				
*DG 18:3, 18:3*	4.66 ± 1.31	3.27 ± 1.41	1.43	0.1988
**Diterperne**				
*Cafestol*	1.43 ± 0.13	0.16 ± 0.10	**8.84**	0
**Flavan-3-ol**				
*Catechin*	65.10 ± 18.64	188.20 ± 9.69	**0.35**	0
**Flavonoids**				
*Hyperoside*	0.12 ± 0.03	0.13 ± 0.03	0.88	0.4182
*Isogentisin*	0.02 ± 0.00	0.03 ± 0.01	0.71	0.1892
*Kaempferol*	0.08 ± 0.02	0.08 ± 0.01	0.89	0.4122
*Naringin*	0.01 ± 0.00	0.03 ± 0.03	0.38	0.1868
*Quercetin*	0.03 ± 0.01	0.01 ± 0.00	**3.43**	0.0269
*Quercetin-3-O-glucoside*	0.56 ± 0.07	1.40 ± 0.06	**0.4**	0
*Quercitrin*	24.33 ± 1.60	11.91 ± 0.96	**2.04**	0
*Rutin*	0.08 ± 0.04	0.15 ± 0.04	0.58	0.0616
**Phenolic acids**				
*3,4,5-Trihydroxycinnamic acid*	0.13 ± 0.06	0.04 ± 0.02	**3.55**	0.0241
*3,4-Dimethoxycinnamic acid*	0.13 ± 0.03	0.14 ± 0.03	0.92	0.5788
*3,4,5-Trimethoxycinnamic acid*	0.05 ± 0.02	0.03 ± 0.01	1.84	0.0791
*Caffeic acid*	0.16 ± 0.05	0.14 ± 0.05	1.16	0.539
*Cinnamic acid*	0.41 ± 0.05	0.68 ± 0.05	**0.61**	0.0002
*Coumaric acid*	0.04 ± 0.01	0.03 ± 0.01	1.18	0.2813
*Ferulic acid*	0.19 ± 0.05	0.42 ± 0.05	**0.46**	0.0008
*Gallic acid*	0.03 ± 0.01	0.00 ± 0.00	**0**	0
*Sinapic acid*	0.04 ± 0.01	0.06 ± 0.03	0.7	0.2638
*Syringic acid*	0.05 ± 0.02	0.00 ± 0.00	**0**	0
*Vanillic acid*	0.15 ± 0.03	0.20 ± 0.04	0.75	0.0894
**Phospholipids**				
*PC 16:0*	53.61 ± 4.42	0.00 ± 0.00	**0**	0
*PC 18 2*	61.63 ± 8.12	127.55 ± 9.26	**0.48**	0
*PC 18:1*	7.35 ± 0.91	10.02 ± 1.18	**0.73**	0.0114
*PE 18:0*	81.71 ± 8.04	91.67 ± 3.10	0.89	0.0602
*PI 16:0*	57.55 ± 5.08	66.38 ± 4.41	**0.87**	0.0394
*PS 17:1*	13.17 ± 1.93	0.00 ± 0.00	0	0
*PS 21:0*	9.79 ± 0.46	12.73 ± 0.55	**0.77**	0.0002
**Chlorogenic acids**				
*1,4-Dicaffeoylquinic acid*	0.47 ± 0.07	0.69 ± 0.01	**0.68**	0.0009
*3-Caffeoyl-4-feruloylquinic acid*	6.44 ± 0.36	16.37 ± 0.96	**0.39**	0
*3-O-Caffeoyl-γ-quinide*	14.12 ± 3.51	39.74 ± 3.02	**0.36**	0
*3-O-Caffeoylquinic acid*	46.20 ± 5.10	95.10 ± 2.78	**0.49**	0
*3-O-Dimethoxycinnamoyl-4-O-quinic acid*	0.16 ± 0.02	0.26 ± 0.04	**0.62**	0.0046
*3-O-Dimethoxycinnamoylquinic acid*	0.31 ± 0.19	0.28 ± 0.03	1.12	0.7396
*3-O-Feruloylquinic acid*	1.18 ± 0.23	2.95 ± 0.26	**0.4**	0.0001
*3-O-p-Coumaroyl-4-O-caffeoylquinic acid*	0.22 ± 0.01	0.94 ± 0.18	**0.24**	0.0002
*3,5-Dicaffeoylquinic acid*	247.07 ± 12.43	474.24 ± 68.40	**0.52**	0.0006
*4-Caffeoyl-5-feruloylquinic acid*	2.55 ± 0.10	6.76 ± 0.40	**0.38**	0
*4-O-Caffeoylquinic acid*	966.22 ± 63.02	1343.81 ± 68.85	**0.72**	0.0002
*4-O-Feruloylquinic acid*	67.48 ± 5.45	104.13 ± 10.47	**0.65**	0.0008
*4,5-Dicaffeoylquinic acid*	247.21 ± 12.30	518.56 ± 22.23	**0.48**	0
*5-O-Caffeoyl-muco-γ-quinide*	1.11 ± 0.06	0.79 ± 0.03	**1.41**	0.0001
*5-O-Caffeoylquinic acid*	4.96 ± 0.45	5.03 ± 0.29	0.99	0.8145
*5-O-Coumaroylquinic acid*	61.29 ± 3.29	83.67 ± 6.64	**0.73**	0.0009
*Feruloyl-1,5-quinide lactone*	0.09 ± 0.03	0.06 ± 0.01	1.64	0.0973
**Sugars**				
*Arabinose*	2.06 ± 0.56	3.18 ± 0.80	0.65	0.0617
*Glucose*	122.05 ± 7.17	166.72 ± 12.13	**0.73**	0.0007
*Raffinose*	1.37 ± 0.28	1.45 ± 0.44	0.95	0.7821
*Stachyose*	0.00 ± 0.00	0.17 ± 0.06	**0**	0
*Sucrose*	30.05 ± 3.47	98.30 ± 8.03	**0.31**	0
**Vitamins**				
*α-tocopherol*	0.08 ± 0.04	0.06 ± 0.02	1.36	0.3491
*Nicotinic acid*	5.23 ± 0.30	5.02 ± 0.39	1.04	0.4296
*Pyridoxine*	0.96 ± 0.17	1.60 ± 0.14	**0.6**	0.0012
*Riboflavin*	0.33 ± 0.04	0.30 ± 0.03	1.13	0.1755
*Thiamine*	0.36 ± 0.06	0.17 ± 0.03	**2.19**	0.001

Data in red indicate higher content in the cascara Arabica; data in green indicates higher content in the cascara Garnica. Only the data with *p*-value 0.05 are highlighted. Four measurements were made per coffee cascara sample (Supplementary Table S1). Abbreviations: DG, diglyceride; PC, phosphatidylcholine; PE, phosphatidylethanolamine; PI, phosphatidylinositol; PS, phosphatidylserine.

**Table 2:** Main molecules identified in coffee by-products using MS-DART-TOF positive/negative mode.

Putative Molecule	Plant Material	Mode	*m*/*z*	Precursor Ion/Adduct	R.I. (%)	Others *m*/*z* [53,54,55]	Molecule Weight	Molecule Formula (M)	Chemical Class
*Caffeine*	*Cascara* *Silverskin Parchment* *Green coffee*	(+)	195.14 195.08 195.08 195.08	[M + H]^+^	100 100 100 100	285, 171, 117	194.16	C_8_H_10_N_4_O_2_	Alkaloid
*Caffeoylquinic acid*	*Silverskin* *Parchment*	(+)	355.20	[M + H]^+^	-- -- -- --	193, 181, 175, 163, 157, 147, 145, 139, 135, 129, 117	354.31	C_16_H_18_O_9_	Chlorogenic acid
*Caffeic acid*	*Cascara* *Silverskin* *Parchment* *Green coffee*	(+)	181.14 181.08 181.08 181.08	[M + H]^+^	14–3 15–5 16–6 2.5–1	163, 145, 135, 117	180.16	C_9_H_8_O_4_	Phenolic acid
*Quinic acid*	*Cascara* *Silverskin* *Parchment*	(+)	193.13 193.08 193.08	[M + H]^+^	5.6–2 7–3 11.5–8	175, 157, 147, 139, 129	192.17	C_7_H_12_O_6_	Carboxylic acid
*Glucose*	*Cascara* *Silverskin* *Parchment* *Green coffee*	(+)	198.14 198.09 198.09 198.09	[M + NH_4_]^+^	18–1 55–39 80–38 0.95	180, 163, 145, 127	180.16	C_6_H_12_O_6_	Sugar
*Quercetin*	*Parchment*	(+)	303.12	[M + H]^+^	10–26	285	302.23	C_15_H_10_O_7_	Flavonoid
*Caffeine*	*Cascara* *Silverskin* *Parchment* *Green coffee*	(−)	179.06 179.05 179.05 179.05	[M-CH_3_]^−^	13–10 20–41 100–38 100–48	387, 283, 255, 237, 209, 193	194.16	C_8_H_10_N_4_O_2_	Alkaloid
*Caffeoylquinic acid*	*Cascara* *Silverskin* *Parchment* *Green coffee*	(−)	191.06 191.05 191.05 191.05	[M-Caffeoyl]^−^	31–15 14–3 7–2 100–60	353, 179, 173, 135	354.31	C_16_H_18_O_9_	Chlorogenic acid
*Dicaffeoylquinic acid*	*Green coffee*	(−)	533.18	[M + OH]^−^	18–16	489, 371, 353, 335, 191	516.4	C_25_H_24_O_12_	Chlorogenic acid
*Glucose*	*Cascara* *Silverskin* *Parchment* *Green coffee*	(−)	215.04 215.03 215.03 215.03	[M + Cl]^−^	100–80 100 100 58–30	359	180.16	C_6_H_12_O_6_	Sugar

## Data Availability

The data presented in this study are available on request from the authors. The raw data supporting the conclusions of this article will be made available by the authors on request.

## Supplementary Materials

The following supporting information can be downloaded at: https://www.mdpi.com/article/10.3390/plants13192741/s1, Supplementary Word File, Figure S1: HPLC-ESI (+/−)-HRMS profiles of cascara Arabica and cascara Garnica samples. A. TIC (Total Ion Current) of negative ionization mode profiles; B. TIC of positive ionization mode profiles; Table S1: Data HPLC-ESI-HRMS. Four measurements were made per coffee husk sample. Arabica (A1–A4), Garnica (B1–B4); Figure S2: DART mass spectra of coffee by-products and green coffee in positive and negative ion mode.
