# Supplementary material for: Bioactive Compounds and Valorization of Coffee By-Products from the Origin: A Circular Economy Model from Local Practices in Zongolica, Mexico
Source: Plants (Basel). 2024 Sep 30;13(19):2741. doi: 10.3390/plants13192741 (PMC11478550; doi:10.3390/plants13192741)
Supplement: Supplementary file 1 [file plants-13-02741-s001.zip › plants-3161643-supplementary.pdf]

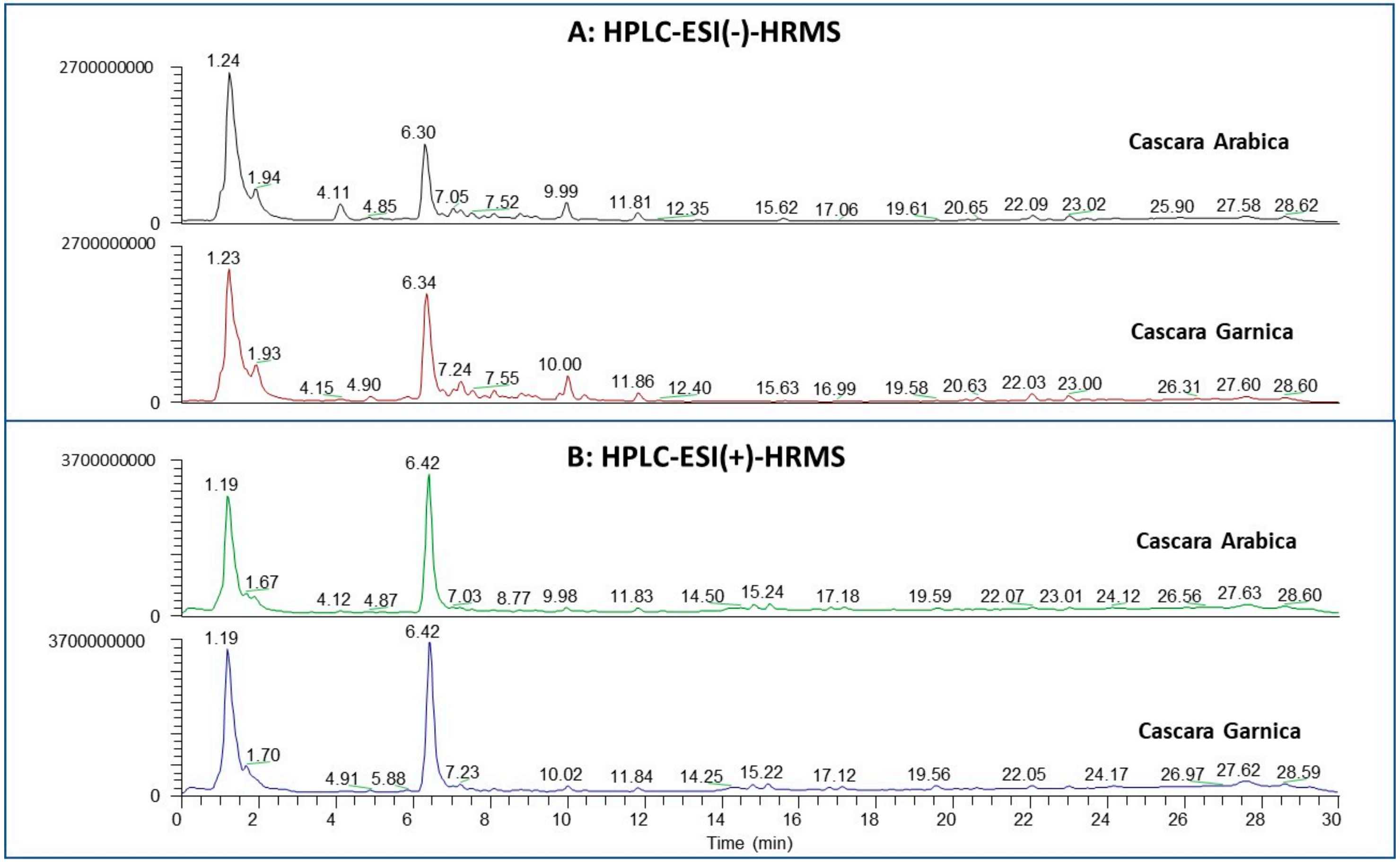

Figure S1: HPLC-ESI (+/-)-HRMS profiles of Cascara Arabica and Cascara Garnica samples. A. TIC (Total Ion Current) of negative ionization mode profiles; B. TIC of positive ionization mode profile

Table S1. Data HPLC-ESI-HRMS. Four measurements were make per coffee husk sample. Arabica (A1-A4), Garnica (B1-B4)

Metabolic class	Metabolite	A1	A2	A3	A4	B1	B2	B3	B4
Acid	Citraconic acid	0.14	0.18	0.18	0.13	0.08	0.11	0.11	0.09
Acid	Glutaric acid	8.71	9.49	11.05	10.28	1.94	2.22	1.70	1.78
Acid	Glycolic acid	0.53	0.48	0.56	0.48	0.42	0.42	0.51	0.53
Acid	Itaconic acid	0.28	0.44	0.50	0.35	0.38	0.41	0.44	0.48
Acid	Lactic acid	126.94	131.29	129.72	115.14	166.32	183.09	168.12	181.70
Acid	Malic acid	0.16	0.28	0.25	0.16	0.10	0.16	0.22	0.03
Acid	Oxalic acid	0.03	0.02	0.09	0.01	0.15	0.15	0.16	0.27
Acid	Pyruvic acid	15.17	15.95	16.65	15.17	16.11	16.14	20.27	21.99
Acid	Quinic acid	1029.84	1124.25	951.20	901.20	757.80	762.68	800.17	875.88
Acid	Shikimic acid	3.00	2.55	2.75	2.53	1.08	0.91	0.82	0.80
Acid	Succinic acid	1.60	1.69	2.17	1.94	1.13	1.05	1.10	1.35
Acid	Tartaric acid	1.98	1.56	2.18	1.63	0.23	0.25	0.51	0.48
Alkaloid	Caffeine	4505.10	4825.05	4865.39	4354.16	4997.41	4999.09	5032.85	5310.18
Alkaloid	Serotonin	0.06	0.06	0.01	0.02	0.24	0.24	0.32	0.18
Alkaloid	Theobromine	9.91	10.68	9.19	8.22	6.97	7.06	7.61	8.10
Alkaloid	Theophylline	10.44	10.85	10.39	9.32	2.38	2.28	2.39	2.61
Alkaloid	Trigonelline	0.01	0.00	0.01	0.01	0.00	0.01	0.03	0.01
Amino acid	4-Hydroxyproline	0.85	0.74	1.04	0.76	0.88	0.75	0.99	1.00
Amino acid	Arginine	0.41	0.24	0.44	0.44	0.10	0.24	0.17	0.12
Amino acid	Asparagine	12.79	13.38	13.67	11.60	7.00	6.78	6.93	7.13
Amino acid	Aspartic acid	6.07	6.98	7.69	6.94	11.56	12.08	16.56	17.81
Amino acid	Glutamic acid	2.99	1.73	6.97	5.02	5.07	4.89	5.85	5.18
Amino acid	Glutamine	0.45	0.40	0.39	0.43	0.32	0.35	0.40	0.37
Amino acid	Histidine	0.06	0.12	0.03	0.05	0.11	0.12	0.11	0.14
Amino acid	Leucine-Isoleucine	1.46	1.49	1.60	1.44	2.65	2.56	3.29	3.31

Amino acid	Lysine	0.07	0.08	0.11	0.20	0.18	0.17	0.28	0.26
Amino acid	Phenylalanine	4.53	4.77	4.66	4.04	6.58	6.87	7.45	8.35
Amino acid	Pipecolic acid	27.47	28.36	27.56	26.35	22.97	22.66	30.53	32.02
Amino acid	Proline	28.15	2.62	2.52	2.55	18.67	1.48	24.75	26.37
Amino acid	Serine	0.02	0.03	0.01	0.02	0.02	0.04	0.02	0.01
Amino acid	Threonine	0.11	0.11	0.09	0.10	0.06	0.09	0.11	0.12
Amino acid	Tryptophan	2.72	2.90	1.83	1.56	0.09	0.09	0.12	0.12
Amino acid	Tyrosine	4.53	4.90	4.74	4.06	2.78	3.41	3.36	3.86
Amino acid	Valine	31.01	33.14	45.50	41.80	11.98	11.88	14.80	15.86
Diglyceride	DG 18:3, 18:3	2.98	5.21	6.06	4.38	5.32	2.33	2.32	3.09
Diterpene	Cafestol	1.47	1.58	1.38	1.27	0.07	0.07	0.24	0.26
Fatty acid	Adipic acid	0.86	0.31	0.98	0.75	0.66	0.89	0.65	0.29
Fatty acid	Arachidic acid	22.04	22.64	24.73	22.78	21.28	23.18	24.31	27.41
Flavan-3-ol	Catechin	76.89	84.97	51.35	47.21	179.40	182.91	189.05	201.45
Flavonoid	Hyperoside	0.08	0.12	0.12	0.14	0.12	0.17	0.14	0.11
Flavonoid	Isogentisin	0.02	0.03	0.02	0.03	0.04	0.02	0.03	0.04
Flavonoid	Kaempferol	0.07	0.09	0.05	0.09	0.10	0.08	0.07	0.09
Flavonoid	Naringin	0.01	0.01	0.01	0.01	0.06	0.01	0.01	0.04
Flavonoid	Quercetin	0.02	0.02	0.03	0.05	0.01	0.01	0.01	0.01
Flavonoid	Quercetin-3-O-glucoside	0.59	0.62	0.54	0.47	1.40	1.47	1.33	1.40
Flavonoid	Quercitrin	24.34	26.34	24.22	22.43	11.20	11.00	12.53	12.92
Flavonoid	Rutin	0.06	0.09	0.06	0.13	0.20	0.14	0.13	0.11
Phenolic acid	3,4,5-Trihydroxycinnamic acid	0.11	0.05	0.18	0.17	0.01	0.05	0.03	0.05
Phenolic acid	3,4-Dimethoxycinnamic acid	0.14	0.13	0.10	0.16	0.16	0.17	0.13	0.11
Phenolic acid	3,4,5-Trimethoxycinnamic acid	0.08	0.06	0.03	0.05	0.03	0.03	0.02	0.03
Phenolic acid	Caffeic acid	0.10	0.21	0.16	0.18	0.08	0.15	0.15	0.20
Phenolic acid	Cinnamic acid	0.41	0.45	0.45	0.35	0.68	0.69	0.62	0.73
Phenolic acid	Coumaric acid	0.04	0.04	0.03	0.05	0.04	0.04	0.02	0.03

Phenolic acid	Ferulic acid	0.13	0.23	0.18	0.24	0.36	0.45	0.40	0.48
Phenolic acid	Gallic acid	0.02	0.03	0.02	0.04	nd	nd	nd	nd
Phenolic acid	Sinapic acid	0.03	0.03	0.05	0.04	0.07	0.05	0.02	0.09
Phenolic acid	Syringic acid	0.04	0.08	0.05	0.05	nd	nd	nd	nd
Phenolic acid	Vanillic acid	0.11	0.16	0.16	0.19	0.16	0.19	0.23	0.24
Phospholipid	PC 16:0, 0:0	49.65	52.45	59.94	52.39	nd	nd	nd	nd
Phospholipid	PC 18 2	60.96	73.02	58.48	54.05	128.45	115.01	129.43	137.33
Phospholipid	PC 18:1	7.01	8.66	7.15	6.58	10.33	9.23	8.98	11.55
Phospholipid	PE 18:0	84.25	91.26	79.09	72.26	93.21	91.38	87.47	94.62
Phospholipid	PI 16:0	60.61	62.79	55.15	51.65	70.66	64.44	61.11	69.30
Phospholipid	PS 17:1	12.94	12.55	15.87	11.33	nd	nd	nd	nd
Phospholipid	PS 21:0, 0:0	9.66	10.41	9.32	9.77	12.63	12.36	12.40	13.54
Chlorogenic acid	1,4-Dicaffeoylquinic acid	0.45	0.57	0.47	0.40	0.68	0.69	0.70	0.70
Chlorogenic acid	3-Caffeoyl-4-feruloylquinic acid	6.10	6.30	6.94	6.44	15.57	15.70	16.60	17.63
Chlorogenic acid	3-O-Caffeoyl-γ-quinide	16.44	17.77	11.69	10.59	37.39	38.06	39.42	44.09
Chlorogenic acid	3-O-Caffeoylquinic acid	47.95	52.20	44.39	40.24	93.28	95.98	92.51	98.63
Chlorogenic acid	3-O-dimethoxycinnamoyl-4-O-quinic acid	0.18	0.16	0.14	0.15	0.31	0.25	0.22	0.25
Chlorogenic acid	3-O-Dimethoxycinnamoylquinic acid	0.46	0.50	0.14	0.15	0.27	0.28	0.26	0.32
Chlorogenic acid	3-O-Feruloylquinic acid	1.11	1.51	1.12	0.97	3.06	2.57	3.04	3.14
Chlorogenic acid	3-O-p-Coumaroyl-4-O-caffeoylquinic acid	0.21	0.23	0.24	0.21	0.79	0.79	1.14	1.04
Chlorogenic acid	3,5-Dicaffeoylquinic acid	246.78	262.06	247.79	231.64	416.31	417.03	512.10	551.53
Chlorogenic acid	4-Caffeoyl-5-feruloylquinic acid	2.53	2.50	2.70	2.48	6.50	6.37	6.92	7.26
Chlorogenic acid	4-O-Caffeoylquinic acid	951.42	1044.32	976.95	892.20	1296.56	1295.72	1340.81	1442.14

Chlorogenic acid	4-O-Feruloylquinic acid	68.62	73.52	67.46	60.32	101.86	118.98	94.37	101.34
Chlorogenic acid	4,5-Dicaffeoylquinic acid	246.78	262.19	247.79	232.09	506.39	504.22	512.10	551.53
Chlorogenic acid	5-O-Caffeoyl-muco-γ-quinide	1.09	1.19	1.13	1.05	0.75	0.79	0.78	0.83
Chlorogenic acid	5-O-Caffeoylquinic acid	5.34	5.15	5.05	4.31	4.67	5.27	4.91	5.27
Chlorogenic acid	5-O-Coumaroylquinic acid	59.67	63.43	64.57	57.49	77.44	78.58	87.95	90.69
Chlorogenic acid	Feruloyl-1,5-quinide lactone	0.09	0.12	0.11	0.04	0.06	0.04	0.07	0.06
Sugar	Arabinose	1.78	2.00	2.86	1.60	2.66	3.58	4.11	2.37
Sugar	Glucose	121.17	131.96	120.22	114.85	158.78	155.34	170.80	181.96
Sugar	Raffinose	1.73	1.04	1.37	1.34	1.06	1.50	1.18	2.04
Sugar	Stachyose	nd	nd	nd	nd	0.25	0.14	0.10	0.18
Sugar	Sucrose	30.28	34.86	27.90	27.18	91.56	92.47	100.39	108.77
Tocochromanol	α-tocopherol	0.03	0.09	0.11	0.09	0.07	0.05	0.04	0.08
Vitamin	Nicotinic acid	4.99	4.97	5.58	5.37	4.67	4.76	5.53	5.11
Vitamin	Pyridoxine	1.00	0.73	0.99	1.13	1.40	1.60	1.67	1.72
Vitamin	Riboflavin	0.31	0.33	0.38	0.31	0.28	0.26	0.33	0.32
Vitamin	Thiamine	0.35	0.33	0.45	0.33	0.18	0.12	0.18	0.20

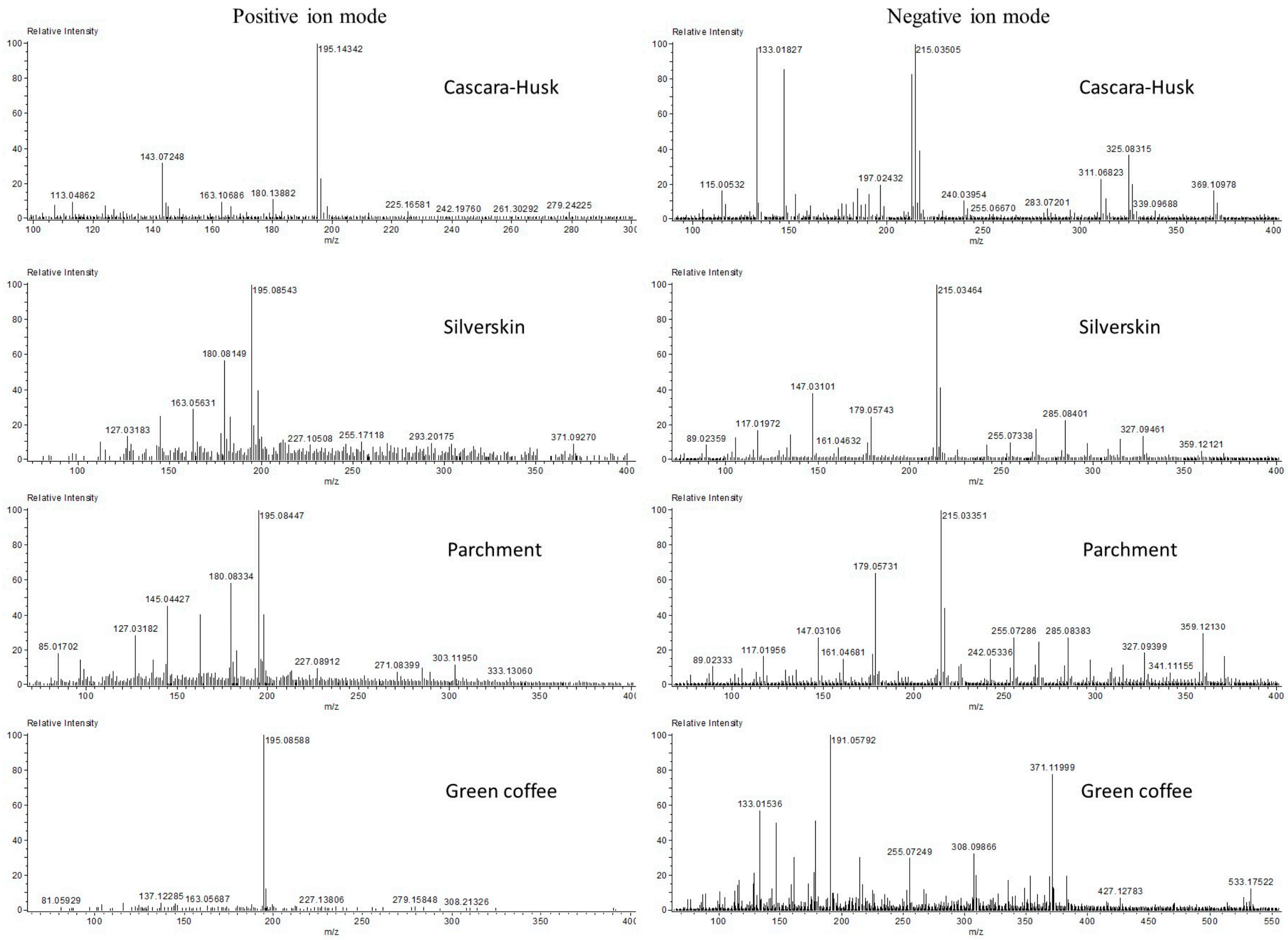

Figure S2. DART mass spectra of coffee by-products and green coffee in positive and negative ion mode
